# Probing the effect of OSCE checklist length on inter-observer reliability and observer accuracy

**DOI:** 10.3402/meo.v20.29242

**Published:** 2015-10-20

**Authors:** Katrina F. Hurley, Nick A. Giffin, Samuel A. Stewart, Graham B. Bullock

**Affiliations:** 1 Department of Emergency Medicine, Dalhousie University, Halifax, NS, Canada; 2 Bachelor of Medicine Class of 2016, Dalhousie University, Halifax, NS, Canada; 3 Division of Medical Informatics, Dalhousie University, Halifax, NS, Canada

**Keywords:** medical education, assessment, OSCE, reliability, metrics

## Abstract

**Purpose:**

The Objective Structured Clinical Examination (OSCE) is a widely employed tool for measuring clinical competence. In the drive toward comprehensive assessment, OSCE stations and checklists may become increasingly complex. The objective of this study was to probe inter-observer reliability and observer accuracy as a function of OSCE checklist length.

**Method:**

Study participants included emergency physicians and senior residents in Emergency Medicine at Dalhousie University. Participants watched an identical series of four, scripted, standardized videos enacting 10-min OSCE stations and completed corresponding assessment checklists. Each participating observer was provided with a random combination of two 40-item and two 20-item checklists. A panel of physicians scored the scenarios through repeated video review to determine the ‘gold standard’ checklist scores.

**Results:**

Fifty-seven observers completed 228 assessment checklists. Mean observer accuracy ranged from 73 to 93% (14.6–18.7/20), with an overall accuracy of 86% (17.2/20), and inter-rater reliability range of 58–78%. After controlling for station and individual variation, no effect was observed regarding the number of checklist items on overall accuracy (*p*=0.2305). Consistency in ratings was calculated using intraclass correlation coefficient and demonstrated no significant difference in consistency between the 20- and 40-item checklists (ranged from 0.432 to 0.781, *p*-values from 0.56 to 0.73).

**Conclusions:**

The addition of 20 checklist items to a core list of 20 items in an OSCE assessment checklist does not appear to impact observer accuracy or inter-rater reliability.

Since its inception in the 1970s, the Objective Structured Clinical Examination (OSCE) has been widely employed as a means of assessing clinical competence (1). The OSCE is an assessment of specific medical competencies in a simulated environment as a proxy for true clinical competence using a criterion-based checklist or global rating scale (2–4). This tool strives to meet the characteristics of an ideal assessment method by maximizing validity, reliability, objectivity, and feasibility (5). It is widely used in the summative assessment of practical competencies for undergraduate medical students.

The OSCE can be used to assess multiple dimensions of student performance including: history taking, physical examination, communication, procedural skills, problem solving, and decision-making (2). However, the drive to assess multiple constructs within a single encounter may lead to highly complex OSCE checklists. Complexity may impact the accuracy of the assessment through: 1) trivialization of the checklist, whereby the required task is broken down into a detailed list, no longer reflecting the task as a whole (6); 2) observer overload, which may negatively impact rating behavior, as measured by reduced inter-observer reliability and reduced observer accuracy when compared to a gold standard (7, 8); and 3) poor checklist design and execution, increasing the likelihood of dysfunctional and/or inaccurate assessment, potentially masking decreased validity with seemingly appropriate inter-observer reliability.

The reliability and validity of the OSCE have been studied through various lenses including well-characterized psychometric effects (2). For example, its validity has been demonstrated using the construct of experience, in that more experienced residents perform at a higher level than junior residents who, in turn, outperform medical students (9–11). However, we contend the effect of checklist length on observer accuracy has been understudied. One such investigation could be identified which directly examined the effect of checklist length on observer accuracy. In this study, Vu et al. demonstrated a statistically significant relationship between observer accuracy and checklist length when standardized patients were the observers (12). However, checklist length varied from as few as five items, up to maximum of 30 items. Considering the high stakes nature of the OSCE, observer accuracy is clearly worthy of further exploration.

The objective of this study was to determine whether increasing the length, and thereby complexity, of an OSCE checklist would have a measurable effect on inter-observer reliability and observer accuracy as judged against a predetermined gold standard. We hypothesized that inter-observer reliability and observer accuracy would diminish as the number of checklist items increased.

## Methods

### Human subjects review

Institutional research ethics committees approved this study; REB form number CD-2003-197.

### Development of OSCE scenarios

In cooperation with Dalhousie University's Learning Resource Centre, four OSCE scenarios with corresponding 40-item checklists were developed to assess the performance of a senior medical student (clinical clerk). Each scenario was designed as a 10-min integrated history and physical examination OSCE station using a standardized patient. A volunteer medical student ‘examinee’ enacted four scripted scenarios with standardized patients and was video recorded. The encounter was scripted to reflect a balance of both satisfactory and unsatisfactory performance on checklist items. Video recording was used in this study to minimize the variability inherent to simulated experiences involving multiple participants, and isolate variations to the observer.

### Development of checklists

To set the ‘gold standard,’ a panel of four emergency physicians (KFH, GB, and two other experienced academic physicians) reviewed the recorded interactions repeatedly. We coded the checklist items as binary outcomes, either satisfactorily or unsatisfactorily completed. Disagreements on specific items were resolved by discussion and repeated video review until a consensus could be reached. The panel consensus score on each item was taken as the standard measurement against which subsequent assessments were compared.

Each physician in the panel was also asked to rank the checklist items in order of importance. The 20 most highly ranked items were deemed to be ‘core’ and comprised the 20-item checklist. The remaining 20 items were integrated into the core lists, generating the 40-item checklists to determine the accuracy of the participants’ assessment of a 20-question checklist when confounded by the integration of 20 additional criteria. The checklist was designed to be completed by a physician observer rather than a standardized patient.

### Participants

Study participants included staff physicians in the Emergency Departments of the QEII Health Sciences Centre and Saint John Regional Hospital sites as well as senior residents (postgraduate years 3–5) in Emergency Medicine at Dalhousie University during 2002–2005. No specific training was provided prior to participation. These individuals were chosen as a convenience sample of reviewers. Participants had not previously viewed the video. Those individuals involved in station development and standardization were excluded from further participation. Study subjects did not receive any financial compensation for participating.

### Participant recruitment

Eligible physician subjects were approached for informed consent by the principal investigator. Physicians who agreed to participate were given verbal instructions and a sealed envelope including the 40-min video (four 10-min scenarios), four corresponding checklists and a letter instructing them to view the video only once, while sequentially completing the checklists. Study packages were assembled in advance of recruitment, with random combinations of two 20-item and two 40-item checklists. Subjects scored each checklist item based on their observation of the videotaped interactions. The scored checklists were placed into a sealed envelope and returned to the principal investigator. Data were stored in a locked cabinet and not examined until the end of the study period. No identifying data were recorded on study participants.

### Data management and analysis

The accuracy of the participants was determined as the proportion of correctly scored core items as compared to the ‘gold standard’ panel consensus. An *a priori* decision was made that a 5% difference in observer accuracy between the 20- and 40-item checklists would be considered academically significant. The data were analyzed using R version 2.15.2 (13). The individual checklist data were used to calculate student grades (the observer's assessment of the student), observer accuracy (the assessment of the rater as compared to the ‘gold standard’), and inter-rater reliability (the degree of agreement between raters). The effect of the number of items on observer accuracy and student grades was evaluated using multiple linear regressions, controlling for individual observer and scenario variation. Inter-rater reliability was calculated using one-way intraclass correlations coefficient (ICC) and tested using an *F*-test (14).

## Results

**Fig. 1:**
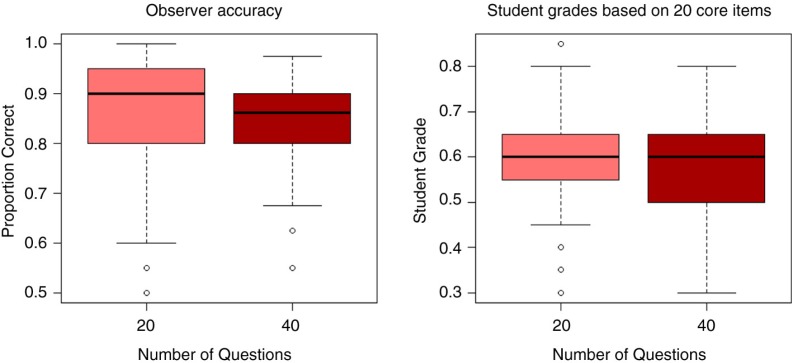
Boxplots of observer accuracies and student grades.

Fifty-seven physician observers completed 228 assessment checklists. The distribution of observer accuracies and student grades is shown in Table 1 and Fig. 1.

**Table 1:** Distribution of observer accuracy and student grades for four OSCE stations as mean raw scores (% mean)

Station	20-item (accuracy^a^)	40-item (accuracy^a^)	20-item (grade^b^)	40-item (grade^b^ on core)	40-item (grade^b^ on all)
Abdominal pain	17.4 (87%)	33.5 (84%)	12.3 (62%)	11.4 (57%)	22.7 (57%)
Jaundice	18.2 (91%)	35.8 (90%)	12.4 (62%)	11.8 (59%)	23 (57%)
Knee pain	18.6 (93%)	35.6 (89%)	13.8 (69%)	14.1 (70%)	24.2 (61%)
Vertigo	14.3 (72%)	30.3 (76%)	10.2 (51%)	9.5 (48%)	18.9 (47%)

a Accuracy is a comparison of the raters’ scored items and the gold-standard, that is, how close the rater came to the “correct” grades.

b Grade is the final grade on the OSCE that the student would have received from the rater.

The overall mean accuracy of the 20-item lists was 85.6%, compared to 84.5% for the core items on the 40-question lists. A multiple linear regression model was built to control for the variance in accuracy caused by inter-rater variance and individual OSCE station. The model suggests that the 40-item checklists are 1.1% less accurate, a difference that is statistically insignificant (*p*=0.164) and short of the 5% threshold established as the objective of the study. Of note is that both rater and OSCE station had a significant effect on the model, suggesting that different stations had different levels of accuracy (Table 1 and Fig. 1) and that different observers achieved different levels of accuracy. Observers ranged from average accuracies of 72.5–92.2%.

For each of the 80 core checklist items (20 items per station, four stations), the accuracy between the 40-item checklist and the 20-item checklist was tested using a simple *t*-test. The resulting *p*-values ranged from 0.02 to 0.98, none of which met the critical value of *p*<0.003 (using a Bonferroni correction). This result suggests that there were no individual questions whose accuracy was different between the 20- and 40-item checklists.

The inter-observer reliability across checklists was measured using one-way ICC, with values ranging from 0.432 to 0.758 (see Table 2). The number of questions on each checklist did not have a significant effect on the ICC at any of the four stations. The ICC values overall ranged from poor to moderate agreement: the results for the Vertigo station are low, but the rest are acceptable, and all of the stations leave room for improvement.

**Table 2:** Inter-observer reliability, stratified by group and number of questions

	Abdominal pain [95% CI]	Jaundice [95% CI]	Knee [95% CI]	Vertigo [95% CI]
20 Questions	0.673 [0.54, 0.82]	0.758 [0.64, 0.87]	0.723 [0.6, 0.85]	0.542 [0.4, 0.72]
40 Questions – all	0.581 [0.48, 0.7]	0.713 [0.62, 0.81]	0.645 [0.55, 0.75]	0.432 [0.33, 0.56]
40 Questions – core	0.573 [0.43, 0.74]	0.761 [0.64, 0.87]	0.781 [0.67, 0.89]	0.436 [0.3, 0.63]
*p*-Value: 20 vs. 40 (all)	0.655	0.56	0.619	0.724
*p*-Value: 20 vs. 40 (core)	0.668	0.508	0.581	0.716
*p*-Value: 40 (all) vs. 40 (core)	0.515	0.566	0.695	0.509

Table 1 also contains the student grades on both sets of checklists. For the 40-item checklists, grades were calculated in two ways, first using just the 20 core questions and second using all 40 questions. Analyses were restricted to items included in both checklists, as there is no expectation that the full 40-item list should have the same grade as the 20-item list, making comparisons of their grades moot. After controlling for individual observer and station variations using a multiple linear regression, the 40-item checklists were found to have scores 2.3% lower than the 20-item lists (95% CI: [0.4%, 4.1%], *p*=0.017), a statistically significant difference that fell short of the 5% academically significant difference defined in our research protocol.

## Discussion

Understandably, trainee assessment is under constant review (15), including the OSCE (16). While the reliability and validity of the OSCE have been studied more extensively, observer accuracy has been understudied.

Greater objectification, through increasing numbers of checklist items, has been shown to negatively affect reliability with a corresponding trend toward decreased validity (6). Other research has shown that increased rater demands decreased inter-rater reliability and the discriminatory value of an assessment tool using global rating scales (17). No correlation has been found between checklist length and inter-rater reliability (6). Our findings suggest that the addition of 20 items to a consensus ‘core’ list of 20 items reduces the student grade by 2.3% (*p*=0.017) when used by Emergency Physicians to assess student performance in a four-station OSCE after controlling for rater and station variations, which is likely academically insignificant. Since the checklists were designed to be used by a third person observer, the results cannot be generalized to checklists completed by standardized patients. However, Vu et al. identified a negative relationship between checklist length and observer agreement with a regression slope of −0.26 (*χ* ^2^=7.21, *p*=0.007) when standardized patients were the observers (12).

There was no difference in observer accuracy in this four-station OSCE. Given the high stakes nature of many OSCEs, it is surprising to find observer accuracy as low as 73% found in the Vertigo station. Observers appear to err on the side the student, giving the benefit of the doubt and effectively inflating their scores. There is no set benchmark for observer accuracy in OSCEs but less than 90–95% likely would not be considered acceptable for high stakes examinations. Further analysis is required to elucidate the factors effecting overall observer accuracy of a single station and was not the intent of this investigation.

Fifty-seven examiners participated in this study, representing nearly the complete complement of those who were eligible (response rate of 93%). To collect more data, we would have required recruitment of Emergency Physicians from additional health centers, or added more stations and consequently more time to the examination. Lengthening the time commitment for the study may have made recruitment more challenging and, given the similarity of the accuracies between the 20- and 40-item lists, it is unlikely that more data would have unveiled an academically significant 5% difference in accuracy.

### Limitations

Station designs were completed with the assistance of Dalhousie University's Learning Resource Centre who is responsible for planning medical school OSCEs. Many of the study participants had previously acted as examiners in real OSCEs while for others, this represented a novel experience. Although the study participants were not provided with any special training, their scores were closely clustered with few outliers. Furthermore, Newble et al. have shown previously that examiner training is unnecessary for consistent observers and does not affect inconsistent assessments (18). In future studies, it would be interesting to determine the effect of examiner experience on observer accuracy.

The methodology of this investigation is predicated on the observers only reviewing the video a single time. As this process was unsupervised, the potential for repeat viewings exists and could impact the results.

Our conclusions are limited to the number of stations that we tested. It is possible that there may be a significant effect for other types of checklists or other scenarios. The combination of scenarios does, however, represent a constellation of medical complaints representative of those used in an OSCE.

## Conclusions

Addition of 20 items to a consensus, core list of 20 checklist items does not appear to adversely affect observer accuracy or inter-rater reliability in a video reviewed, four-station OSCE. Further analysis is required to elucidate the generalizability of this finding.
